# Intervention for minimizing the risk of drop-out in music school pupils—an exploratory study

**DOI:** 10.3389/fpsyg.2026.1763052

**Published:** 2026-03-04

**Authors:** Ana Kavčič Pucihar, Katarina Zadnik, Brina Zamrnik, Petja Plamberger

**Affiliations:** 1 Academy of Music, University of Ljubljana, Ljubljana, Slovenia; 2 Department of Psychology, Faculty of Arts, University of Ljubljana, Ljubljana, Slovenia; 3 Department of Psychology, Faculty of Arts, University of Maribor, Maribor, Slovenia

**Keywords:** drop out, instrumental music education, intervention, motivation, music theory lessons, self-determination theory

## Abstract

This exploratory study examined an intervention aimed at supporting music school pupils’ motivation and explored its potential relevance for dropout prevention. Drawing on our prior research into motivation and dropout in Slovenian music schools, we developed an intervention to address this problem. We selected three areas previously identified as leading to dropout: *individual differences, challenges with music theory and solfège,* and *limited peer interaction.* We designed 7 workshops to improve pupils’ body posture and motor skills (*Fit Musician*), music learning and instrument practice (*Connected, The Great Practitioner!*), understanding of music theory and solfège (*I Can Master Music!, Super Music Theory!*), and limited peer interaction (*Together is Cute*), along with improvisation skills (*The Impro League*). 55 pupils at a Slovenian public music school, some identified by their teachers as having an elevated risk of dropout, participated in the workshops 8–10 times. These ran mostly alongside their regular curriculum and were led by University of Ljubljana students under the guidance of their teaching and work mentors. We used both qualitative and quantitative data to understand the intervention’s outcomes better. Quantitative data were collected from the pupils (*N* = 43) using adapted motivational questionnaires administered pre-intervention and post-intervention. Qualitative data were collected through focus group interviews with music instrument and music theory teachers (*N* = 13). Quantitative analyses showed a statistically significant increase in pupils’ perceived autonomy (*p* = 0.02) during theory lessons and a statistically significant decrease in perceived competence in instrumental performance (*p* < 0.001). However, the qualitative analysis of their instrumental teachers’ observations revealed increased competence in pupils’ instrumental performance. Furthermore, qualitative analysis revealed several observed shifts in pupils’ motivation. These included changes in relatedness, competence, and positive attitudes toward learning, with more minor changes in autonomy, enthusiasm, and repertoire-driven motivation in music instrument lessons. Theory teachers reported fewer changes, most notably a more positive attitude toward music theory. Our exploratory study’s preliminary findings suggest that structured interventions (e.g., student–mentor workshops) embedded in the ongoing educational process may relate to pupils’ core motivational processes and warrant further investigation as a potential approach to dropout mitigation in music education.

## Introduction

Classical Western music education for children and adolescents worldwide takes many forms, ranging from elementary school music classes, choirs, bands, and orchestras to individual instrumental instruction, which typically occurs in private music studios. But across all these domains, there is a common problem—the steep drop-out. In MacIntyre et al.'s (2017) research on adult musicians, a feedback loop mechanism was identified. In this model, the desire to learn leads adult musicians to increase their effort at learning (i.e., motivational intensity), which fosters the development of perceived competence. This competence, in turn, further enhances their desire to learn, creating a virtuous cycle of motivation for music learning and performance (MacIntyre et al., 2017). Understanding this cyclical motivation is crucial when considering how to foster similar engagement in younger learners. In contrast to the adult-focused findings above, educators and parents face a significant challenge: how to encourage children and adolescents to develop a lifelong involvement with music.

A recent study by Tucker (2024) found that over 50% of students dropped out of music classes after only one year (which included band, chorus, orchestra, and guitar classes). Music educators are undeniably facing incredible difficulties maintaining students for more than one year (Hash, 2021; Tucker, 2024). Evans (2009) supports the claims of the early drop-out problem, as he identifies the first significant surge of dropout at the age of 11, while Ruth and Müllensiefen (2021) state that more than 50% of students drop out of instrumental music by the age of 17, most quitting between the ages of 15 and 17. Research has consistently shown the persistence of this problem, but according to Hash (2021), there has been little consistency in determining why students persist in or drop out of instrumental music education. Music educators cite a lack of commitment to work as the most common reason given for why students drop out (Boyle et al., 1995; Ng and Hartwig, 2011).

Building on our previous literature review on dropout in instrumental music education by Kavčič Pucihar et al. (2024), we now expand it with the latest findings in this field to provide an updated perspective.

In group instrumental education - school band and orchestra programs, the predictive factors include other competing interests and commitments, logistical challenges, and suboptimal students’ social environment (Busch et al., 2012; Cook, 2013; Hash, 2021; Hurley, 2021; Kinney, 2010). A suboptimal social environment encompasses a lack of parental support, inadequate support from parents or teachers, and single-parent families (Cook, 2013; Kinney, 2010; Pitts et al., 2000). Moreover, socioeconomic status—the lower the SES, the higher the dropout rate—is also a strong predictive factor of dropout (Albert, 2006; Busch et al., 2012; Corenblum and Marshall, 1998; Kinney, 2010). Along with SES, Elpus and Abril (2024) identify gender, shared parent/student attendance at outside arts events, and out-of-school arts engagement as significant predictors of both students’ music participation and drop out.

Lower academic achievement is associated with higher dropout rates, as noted in older studies (Gamin, 2005; Kinney, 2010) and some recent ones (Elpus and Abril, 2024; Guhn et al., 2020). Students’ attitude toward their musicianship is shaped by their experiences of satisfaction with instrumental music making (Hash, 2021) and is reflected in their readiness to practice (Cook, 2013; Gamin, 2005), as well as in their loss of motivation to play a musical instrument (Busch et al., 2012; Lowe, 2010). In Lowe’s study (2010) students indicated that the instrument lessons did impact their competence beliefs, and that competence beliefs can, in turn, impact their decisions to continue learning (Lowe, 2010).

Individual studio instruction presents unique challenges compared to group instrumental settings (Gerelus et al., 2017). Additionally, fostering strong musical identities in students is critical for preventing dropout (Gerelus, 2025).

In individual instrumental instruction, compared to persisting students, predictive factors for dropout include: lower motivation and lower achievement (Costa-Giomi, 2004; Costa-Giomi et al., 2005; Gerelus et al., 2017); later onset of playing a musical instrument, lower musical ability, losing interest, preferring other instruments, and lack of practice (Kavčič Pucihar et al., 2024; King, 2016). When students feel supported by their parents and instrumental teachers, their affinity toward practice heightens, and their desire to improve, as well as the prospect of achieving an excellent grade, a challenging and fun repertoire, all contribute to improved motivation to practice (Šulentić Begić et al., 2024). Parental support and a quality music program were significant factors predicting teachers’ perceptions of student persistence levels in instrumental and classroom music (Ng and Hartwig, 2011; Ruth and Müllensiefen, 2021).

In the Slovenian music school context, Kavčič Pucihar et al. (2023, 2024) found that individual differences among students (such as health issues, learning difficulties, and stage fright), unsupportive teaching approaches, non-stimulating classroom environments, uninteresting learning repertoires, and, above all, similar as Sagrillo (2016) found out, mandatory music theory and solfège lessons significantly contribute to dropout.

Understanding motivation is crucial for explaining students’ achievement, performance, well-being, and intentions to continue participation in (or drop out of) music learning throughout school and into adulthood (Evans and Liu, 2019).

The close connection between motivation and dropout in music education is becoming the focus of an increasing number of studies that research both phenomena through the lens of various motivational theories. In the following section, we present key motivational theories relevant to music education, with emphasis on Self-Determination Theory (Ryan and Deci, 2002), which serves as the foundation for the study introduced in the subsequent section.

## Motivational theories in the context of instrumental music education

### Self-determination theory

The majority of motivational studies in instrumental music education draw on Ryan and Deci’s (2002) self-determination theory (Comeau et al., 2015; Evans et al., 2013; Evans and Liu, 2019; Földi and Jozsa, 2022; Földi et al., 2024; Gerelus et al., 2020; Kavčič Pucihar et al., 2024; Schatt, 2018; Utomo, 2025; Wieser et al., 2024; Wieser and Müller, 2025). The psychological needs of competence, autonomy, and relatedness are at the core of self-determination theory. Satisfaction of these needs has considerable explanatory power in educational settings; hence it is the focus of recent attention in music education (Evans and Liu, 2019).

Self-Determination Theory posits that an individual’s motivation to initiate or discontinue engagement in an activity is contingent upon the degree to which three basic psychological needs are satisfied: competence, relatedness, and autonomy. The need for competence refers to the individual’s desire to experience efficacy and mastery in the acquisition and execution of skills. The need for relatedness encompasses the aspiration to establish meaningful social connections and to feel a sense of belonging within a social context. The need for autonomy pertains to the individual’s inclination toward self-determination and volitional control over their actions and decisions (Ryan and Deci, 2002).

Students’ psychological needs satisfaction in the instrumental music teaching process is strongly reflected in their intentions to continue and their practice time (Evans and Liu, 2019). When students feel that their basic psychological needs are not being met, they are more likely to drop out of instrumental lessons (Evans et al., 2013; Kavčič Pucihar et al., 2024; Wieser et al., 2024). When basic psychological needs in the instrumental music classroom are supported through targeted activities that focus on feelings of achievement, knowledge, and affect, intrinsic motivational beliefs develop (Schatt, 2022). Utomo (2025) identified ways for music educators to support students’ basic psychological needs. Autonomy can be supported by providing students a choice, cultivating students’ interests and desires, promoting ownership, and helping students set and achieve goals. Competence can be supported by instilling belief and confidence, developing natural talent, and emphasizing the importance of effort. Relatedness can be supported by creating a safe environment, providing inspiration, promoting teamwork and community, and valuing personal relationships above musical relationships (Utomo, 2025). In Kavčič Pucihar et al. (2024) study, the most important factor for drop out was lack of perceived autonomy, followed by the absence of feelings of competence, and deficiency in relatedness, while positive teacher-student relationships and parental encouragement emerged as essential for student retention. Social relationships have an impact on learning also in instrumental music education settings. Students who make an independent decision to learn their musical instrument have significantly higher intrinsic motivation (Földi and Jozsa, 2022).

Földi et al. (2024) found that for early adolescents, parents exert the greatest influence, whereas teachers and friends play a more significant role for mid-adolescents, and friends become the most decisive influence for late adolescents (Földi et al., 2024). When students participate in group instrumental instruction, such as band, taking additional private lessons with a teacher in a one-to-one setting increases their intrinsic motivation to practice (Schatt, 2018; Schatt, 2022). Autonomous motivation in lessons is negatively associated and controlled motivation is positively associated with the tendency to drop out of music schools (Wieser et al., 2024). Dropout students exhibit less autonomous motivation and stronger amotivation, in addition to a lack of competency (Gerelus et al., 2020). Differences in autonomous motivation exist not only between persistent and dropout students, but they also exist between different nationalities: in Caucasian and Chinese children, for instance, the latter identify more with playing the piano, find it more intrinsically enjoyable, and are more motivated by the desire to please their teachers and parents (Comeau et al., 2015).

Wieser and Müller (2025) identified four motivational profiles in instrumental lessons students: autonomously motivated type (high quality), overall highly motivated type (high quantity), moderately autonomously motivated type (low quality), and a type with poor quantity motivation. The majority of respondents in their study were eager to attend music classes and make music with friends, while the weakest interest was to practice scales, performing in public, taking exams on the instrument and attending music theory classes. The two types with high autonomous motivation preferred most music-related activities significantly more than the other two types. Their study showed differences in the support, provided by instrumental teachers, required to meet music students’ basic psychological needs in music lessons (Wieser and Müller, 2025).

By addressing the factors rooted in SDT (Ryan and Deci, 2002), music educators and policymakers can design interventions and create environments that foster students’ autonomy, competence, and relatedness, thus minimizing the risk of dropping out (Kavčič Pucihar et al., 2024).

Since the SDT (Ryan and Deci, 2002) is most strongly represented in the literature on motivation in music education research, it provides a theoretical framework for the present exploratory study of pupils’ motivation in relation to dropout. It forms a basis for understanding changes in motivation driven by basic psychological needs (competence, relatedness, and autonomy) satisfaction, as well as pupils’ amotivation.

However, some aspects of our exploratory study of the music learning process fall outside the SDT’s (Ryan and Deci, 2002) domain. They can be better understood through the lens of other motivational theories. Attitude toward participation in music activities can be better understood through the motivational theory of planned behavior (Ajzen, 1991; Ajzen, 2011). Enthusiasm in playing a musical instrument can be interpreted through the Positive psychology theory (Seligman and Csikszentmihalyi, 2000), while motivation due to an appealing repertoire in music lessons is in line with Weiner’s (1972) Attribution theory.

### Attribution theory

In his Attribution theory of motivation, Weiner (1972) posits that how people perceive the causes of their successes and failures shapes their future motivation.

Building on this, the theory classifies these causes along three dimensions—locus of control (internal vs. external), stability (stable vs. unstable), and controllability (controllable vs. uncontrollable)—which influence future effort and emotional responses. Asmus (1985) points to teachers’ important role in shaping general music students’ perceived causes of success and failure, which, in turn, affects students’ motivational characteristics. In Schatt's (2011) study, high school band students taking private instrumental studio lessons rated belief statements higher, indicating that private one-to-one instrumental study fosters intrinsic motivation for personal growth.

### Expectancy value theory

Expectancy-value theory has been developed by Jacquelynne Eccles and her colleagues (Eccles et al., 1983; Eccles and Wigfield, 2002; Wigfield and Eccles, 2000). The theory postulates that achievement-related choices are motivated by a combination of people’s expectations for success and subjective task value in particular domains.

According to the expectancy-value model, expectations for success and task value are shaped by various factors. These include child characteristics such as abilities, previous experiences, goals, self-concepts, beliefs, expectations, and interpretations. Environmental influences, including cultural milieu and socializers’ beliefs and behaviors, also play a role.

Sin et al. (2022) highlight the valuable insights that expectancy-value theory offers for a better understanding, particularly in its potential applicability to the teaching and learning of music. Instrumental music teachers must be observant of their students’ competency levels and adjust their instruction accordingly, as this correlates with their students’ motivational levels (Burak, 2014). The transition from primary to secondary school has been identified as an important barrier to student retention. At that point, instrumental music students need particularly strong instructional support from their teacher, as students’ perceived competence beliefs can be just as important as values in determining whether to continue the musical instrument learning (Lowe, 2012), while intrinsic value and utility value are salient predictors of individuals’ intentions to persist in music beyond graduation (Siegal, 2025).

### Theory of planned behavior

According to the theory of planned behavior (Ajzen, 1991; Ajzen, 2011), behaviors are influenced by intentions, which depend on attitudes, subjective norms, and perceived behavioral control. External factors can also force or prevent behaviors, regardless of intention, depending on how much control the individual actually has, and how well perceived control matches actual control.

Building on the theory of planned behavior, the decision to persist in music activities is influenced by several variables. Parental involvement and peer influence are the most influential, while students’ attitudes toward general music classes and their teacher are also significant predictors (Ruybalid, 2016). Students with a positive attitude toward music and a value for music are more likely to continue participating in musical activities even when faced with obstacles (Yoo, 2020).

## Music education in Slovenia and the intervention

To inform readers about the Slovenian music school education system, we will briefly describe it. Although the Slovenian music education system is very specific, there are some similarities with various international types of music education. In Slovenian music schools, children aged 7–15 receive instrumental instruction in the form of individual instruction, also known internationally as private studio instruction or one-to-one tuition, while their ensemble lessons are similar to wind band or choir rehearsals held at schools. Music school is an extracurricular activity, and all the children must pass an entrance exam to enroll (Kavčič Pucihar et al., 2024). The course Music Theory, aimed at developing functional musical literacy, is based on five core activities: solfeggio, performing and interpreting examples from musical literature, creating, listening, and acquiring music-theoretical and formal knowledge. Lessons are conducted in a group format once a week for 45, 60, or 90 min (Zadnik, 2019).

Stemming from recent research on key reasons for dropout in Slovenian music schools (Habe et al., 2024; Kavčič Pucihar et al., 2023, 2024) that identified many factors in line with the SDT (Ryan and Deci, 2002), our exploratory study draws on that theoretical framework in the form of an intervention toward enhancing pupils’ motivation in their music education process.

## Aims of the study

To the best of our knowledge, there have been no interventions specifically designed to reduce dropout in music schools by supporting motivational processes in pupils; therefore, we designed our intervention to answer the question: *What changes in pupils’ motivation occur when we support their basic psychological needs for autonomy, relatedness, and competence in the form of an intervention in an ongoing educational process?*

## Method

### Research design

To research the impact of the intervention, we chose a mixed methods approach (Creswell and Creswell, 2023). We used a sequential mixed-methods design, in which quantitative data were collected from the music school pupils and analyzed first, pre- and post-intervention, followed by qualitative data collection from their instrumental and music theory teachers to help explain and contextualize the quantitative findings (Creswell and Plano Clark, 2011). This approach allowed us to identify emerging patterns and then explore, in greater depth, music school pupils’ experiences and performance in their ongoing music education processes and compare them with their teachers’ observations.

### Participants

In a 4-month intervention during the school year 2024/2025, 55 pupils from the local public music school participated in the quantitative part of the study, encouraged to do so by their parents or teachers. The participants completed two identical questionnaires, one pre-intervention and one post-intervention. Pupils who did not complete both questionnaires were excluded from the analysis. The final sample consisted of 43 pupils with an average age of 10, 37 years (SD = 1.83 years), of whom 33 were female, and 10 were male. The participants played various instruments, namely the oboe, accordion, saxophone, flute, piano, percussion, guitar, violin, viola, cello, baritone, bassoon, and accordion. Participants did not receive any compensation for their participation.

Thirteen teachers from the local public music school participated in the qualitative part of the research (eight instrumental teachers and five music theory teachers). They did not receive any compensation for their participation.

### Description of the intervention workshops

We selected three areas, previously identified as leading to dropout, and designed 7 supplementary workshops to address: *individual differences* (e. g. body posture, motor skills, learning and instrument playing difficulties), *music theory and solfège* (to facilitate understanding of the subject matter), and *limited peer interaction* (additional chamber music sessions and improvisation), that ran supplementary to pupils’ regular music school curriculum, except the *I Can Master Music!* workshop held during regular music theory classes. Pupils attended these 45-min workshops weekly, 8–10 times. The workshops were designed to increase pupils’ perceived feelings of competence, autonomy, and relatedness, as defined in the SDT (Deci and Ryan, 2002).

We named the workshops, so they were easily remembered by pupils: *Fit Musician, I Can Master Music!, Super Music Theory!, Connected, Great Practitioner, Together is Cute*, and *Impro League.*

A student from the Faculty of Health Sciences (Physiotherapy) led the *Fit Musician* workshops, where pupils practiced preventive and corrective exercises to improve proper instrument posture, developed gross and fine motor skills, and engaged in targeted core stabilisation exercises. Students from the Faculty of Education (Special and Rehabilitation Education) and the Academy of Music (Music Education) held the *I Can Master Music!* and *Super Music Theory!* workshops. The *I Can Master Music!* workshop was implemented during regular music theory lessons, as pupils needed ongoing guidance and support for the specific activities, particularly for writing music dictations and consolidating core music-theoretical elements (intervals and scales). The *Super Music Theory!* workshop supplemented the regular music theory lessons, with emphasis on a learner-centered teaching approach. The group activities included musical games, body movement, and creativity, with improvisation on Orff musical instruments and pupils’ chosen instruments. The need for autonomy and competence was fostered through improvisation and individual work, while the need for relatedness was supported through pair and small-group activities. A student from the Academy of Music (Music Art program) led the *Connected* workshops, where pupils were encouraged to transfer their music theory knowledge to their instruments through guided analysis, with emphasis on connecting these two music school curriculum subjects (music theory and instrument). Students from the Academy of Music (Instrumental and Vocal Pedagogy and Music Art programs) held the *Great Practitioner*, *Together is Cute*, and *Impro League* workshops. The *Great Practitioner* workshops were designed to support pupils in their instrument practice, to teach them various practice strategies to implement. Students were guiding the pupils in practicing their regular music school instrument repertoire. The *Together is Cute* workshops were explicitly designed to support pupils’ perceived relatedness and provided opportunities for pupils to play in various chamber groups and to prepare a final concert with the workshop repertoire. Pupils had the autonomy to choose the pieces from the repertoire they liked most. The *Impro League,* in addition to creating opportunities for group music-making, offered pupils the chance to improvise on their instruments in various ways.

Under the guidance of work and teaching mentors, the university students were repeatedly encouraged and guided to interact with the music school pupils in ways that facilitated learning, using approaches that emphasized support for the psychological needs of competence, autonomy, and relatedness, as defined in SDT (Deci and Ryan, 1985).

### Measures

For quantitative data collection, we combined two questionnaires that assessed the same or closely related theoretical constructs and have been used before in educational contexts comparable to the present study. Specifically, we employed the Self-Determination Theory based questionnaire developed for school physical education by Standage et al. (2005), which operationalizes key motivational regulations grounded in SDT. Our choice of this instrument was also based on the structural similarities between physical education and music education, both involving skill acquisition, performance evaluation, sustained practice, and teacher–student interaction. The instrument was adapted linguistically and contextually for use in music schools.

In addition, we used selected subscales of amotivation and intrinsic motivation from the Motivation for Learning Music questionnaire (MLM; Comeau et al., 2019). The MLM was chosen because it was developed explicitly for music-learning contexts and therefore captures domain-specific aspects of students’ motivational experiences that may not be fully addressed by other, more general instruments while maintaining theoretical consistency with SDT.

### The test of self-determination theory in school physical education

The questionnaire on three basic needs in physical education was developed by Standage et al. (2005) to examine students’ motivation to participate in physical education. The Self-Determination Theory (Ryan and Deci, 2002) was chosen as the theoretical basis for motivation. The questionnaire was used to assess the degree to which students’ needs for autonomy, competence, and relatedness were satisfied in physical education classes. The scale measuring the degree of satisfaction with autonomy consisted of five items and was derived from a previous study (Standage et al., 2003), which confirmed its validity. Standage et al. (2005) adopted the competence assessment items from the Intrinsic Motivation Inventory (IMI; McAuley et al., 1989), including 5 items in their instrument. Relatedness was also measured using five items from the Need for Relatedness Scale (Richer and Vallerand, 1998). Psychometric analysis of the questionnaire showed good data model fit, with Cronbach’s alpha coefficients (*α*) ranging from 0,80 to 0,96, confirming the measurement reliability of the scale (Standage et al., 2005).

### The motivation for learning music (MLM)

The Motivation for Learning Music questionnaire is designed to measure the motivation of young musicians (Comeau et al., 2019). It consists of five subscales that measure different aspects of motivation: intrinsic motivation, identification and integration, introjection, external regulation, and amotivation. Each subscale consists of five items. For the purposes of our research, we included the intrinsic motivation and amotivation subscales. The Cronbach’s alpha (α) reliability coefficients for the dimensions used range between 0.85 and 0.87, indicating good internal consistency of the measurement scales. Correlations between the selected subscales and the other variables used by the authors to assess construct validity also provided strong support for the construct (Comeau et al., 2019).

Due to the age of the pupils, we also adapted the response format, replacing the numerical scale from 1 to 7 (1 = strongly disagree, 7 = strongly agree) with seven emoticons that best represented the individual values on the scale. For each item, students had to circle the emoticon that best described how they felt about the statement. An example of an item is:

I learn to play the piano/violin because I enjoy learning new things about music. 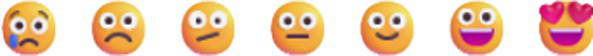


We collected qualitative data through interviews with focus groups of teachers of instruments and music theory whose pupils attended the workshops. We asked the teachers if they had noticed any changes in the motivation of the participating students to attend classes at the local music school.

### Data collection and analysis procedure

We collected data in accordance with the Data Protection Act (Regulation (EU) 2016/679, 2016) and guidelines for ethical research. Before collecting data, we obtained approval from the Research Ethics Committee of the Faculty of Arts at the University of Maribor. Participation was entirely voluntary, and participants could withdraw at any time without consequences. Before data collection began, both teacher and pupil participants (or, in the case of minors, their parents) signed an informed consent form to participate. To ensure anonymity, we excluded all information that could identify participants during data collection.

We collected quantitative data twice, once pre-intervention and once post-intervention. We organized it in Microsoft Excel and processed it statistically using IBM SPSS Statistics, version 29. First, we cleaned the database, identified any missing values, coded the variables appropriately, and summed the items that comprised the individual dimensions. When determining the normality of the distribution of variables, we initially relied on the Shapiro–Wilk test. We also assumed that the variables were normally distributed if the asymmetry and kurtosis coefficients were within the range (−2, 2) (George and Mallery, 2010). Both criteria yielded mixed results, as some variables met the assumption of normality, while others did not. Based on the test results, we decided to use a nonparametric test, specifically the Wilcoxon signed-rank test. The use of nonparametric tests can be further justified by the finding that normality tests are unreliable for small samples, as they are not sensitive to all significant deviations from normality, which makes nonparametric tests more methodologically justified (Field, 2013).

For all tests, we additionally calculated the effect sizes using the following equation.

$$r=\frac{Z}{\sqrt{N}}$$

Here, *r* represents effect size, *Z* statistic, and *N* the sum of negative and positive ranks.

We collected qualitative data through Zoom focus groups. We had two focus groups - one for the music instrument teachers (*N* = 8) and the other for the music theory teachers (*N* = 5). We had one interview with each focus group after the intervention was completed. The prompts were the questions for the teachers, namely: *“What were your observed changes in your pupils’ motivation to pursue their educational process in the music school?”* We recorded the conversations and used MacWhisper (version 11, Bruin, 2024) to create transcripts, which we then manually coded, analyzed for content, and grouped into codes, categories, and themes. The categorization of responses was carried out in pairs of authors, and the final categorization was reviewed and evaluated by the entire research group, thereby satisfying the requirements for intercoder reliability. Data categorization by topic was carried out in two steps. First, we identified the thematic areas of the responses, and in the second step, we assessed the placement of the themes within existing theoretical constructs. The coding framework was based on items from Self-Determination Theory (Ryan and Deci, 2002), while the content analysis also identified items from the Theory of Planned Behavior (Ajzen, 2011), the Positive psychology theory (Seligman and Csikszentmihalyi, 2000), and Weiner’s (1972) Attribution theory. The data were categorized and coded in pairs by the authors and then checked by all four authors to ensure consistency among the evaluators.

We decided to use both qualitative and quantitative data to provide a clearer picture of the possible effects of our intervention from both pupils’ and teachers’ perspectives.

## Results

### Quantitative results

#### Descriptive statistics

To obtain a broader overview of our data, we conducted descriptive statistics for all relevant variables (see Appendix 1).

Descriptive statistics for all variables are presented in Appendix 1. Autonomy in the instrument group showed mean values of 30.07 (SD = 6.92; range = 18–42) at pre-intervention and 28.95 (SD = 6.71; range = 15–42) at post-intervention, while competence had mean values of 29.76 (SD = 5.26; range = 10–35) and 26.36 (SD = 4.09; range = 15–35), and relatedness had mean values of 28.86 (SD = 6.14; range = 12–35) and 29, 50 (SD = 4.09; range = 10–35). In the musical theory group, autonomy averaged 17.61 (SD = 6.19; range = 6–36) at pre-intervention and 21.11 (SD = 7.78; range = 9–42) at post-intervention, competence averaged 23.02 (SD = 7.12; range = 7–35) and 23.24 (SD = 6.67; range = 9–35), and relatedness averaged 27.07 (SD = 7.74; range = 6–35) and 28.40 (SD = 6.73; range = 9–35). Intrinsic motivation in the instrument group showed mean values of 31.21 (SD = 4.06; range = 21–35) at pre-intervention and 31.18 (SD = 4.57; range = 19–35) at post-intervention, while amotivation had mean values of 7.23 (SD = 3.80; range = 5 − 23) and 8.40 (SD = 5.99; range = 5–29), respectively. In the musical theory group, intrinsic motivation averaged 21.10 (SD = 10.22; range = 18–42) at pre-intervention and 22.27 (SD = 10.10; range = 5–35) at post-intervention, and amotivation averaged 14.65 (SD = 9.42; range = 5–35) and 17.25 (SD = 10.70; range = 5–35).

Examination of skewness and kurtosis values shows that several variables deviated from normality, most notably amotivation measures. Intrinsic motivation, autonomy, relatedness and competence showed milder deviations from normality, although Shapiro–Wilk tests were significant for most variables, especially the motivational scales (Appendix).

#### Changes

To compare the results on individual dimensions pre-intervention and post-intervention, we used the Wilcoxon signed-rank test. Additionally, we calculated the effect sizes of all comparisons. In the following, we will report the results separately by questionnaire content and learning context, divided into instrument lessons and music theory lessons (Table 1).

**Table 1:** Changes in autonomy, competence and relatedness in music theory lessons and instrument classes.

Subject	Variable	*Z*	*p*	Effect size
Music theory lessons	Autonomy - pre- and post-intervention	**–2.26**	**0.02**	**0.36**
	Competence - pre- and post-intervention	−0.74	0.46	0.12
	Relatedness - pre- and post-intervention	−0.76	0.45	0.13
Instrument class	Autonomy - pre- and post-intervention	−1.23	0.22	0.20
	Competence - pre- and post-intervention	**−4.06**	**< 0.001**	**0.65**
	Relatedness - pre- and post-intervention	−0.55	0.58	0.09

Bold values state statistically significant changes in values.

For most variables, the test did not record statistically significant changes in values pre-intervention and post-intervention. We recorded statistically significant changes in two cases. In the context of music theory classes, we measured a statistically significant change in values on the autonomy dimension. The mean values on the autonomy scales in Appendix 1 showed that values rose after the intervention ended. Within instrument classes, significant changes occurred in the competence dimension. Upon reviewing the mean values in Appendix 1, we observed that the competence scale mean decreased after the intervention. In both cases, we observed medium to large effect sizes (Field, 2013), indicating that these findings reflect effects of moderate to large magnitude, in contrast to the predominantly small effects observed in the remaining analyses. We did not measure any statistically significant changes in motivation to learn an instrument in any case (Table 2).

**Table 2:** Changes in motivation to learn in music theory lessons and instrument classes.

Subject	Variable	*Z*	*p*	Effect size
Music theory lessons	Amotivation - pre- and post-intervention	−1.39	0.16	0.25
	Intrinsic motivation - pre- and post-intervention	−0.69	0.49	0.12
Instrument class	Amotivation - pre- and post-intervention	−0.53	0.60	0.11
	Intrinsic motivation - pre- and post-intervention	−0.22	0.83	0.04

We measured no statistically significant changes in the dimensions of motivation pre-intervention and post-intervention, both in music theory lessons and in instrument classes. Observed effects were predominantly small.

### Qualitative results

The analysis of focus group data with instrumental teachers (Table 3) and music theory teachers (Table 4) yielded seven themes: autonomy, relatedness, competence, amotivation, attitude change, enthusiasm, and increased motivation due to appealing repertoire. These themes categorized the observed changes in pupils’ motivation to continue their musical education at the music school. For a detailed description of each theme and associated codes, refer to Tables 3, 4.

**Table 3:** Observed changes in pupils’ motivation in music instrument lessons by their instrument teachers.

Theme	Category	Code
Autonomy	Intrinsic motivation	Independent practice without external prompting (1)
Relatedness	Relatedness among peers	Physical closeness and shared activity (1)
		Peer bonding and new friendships (2)
		Peer support (1)
		Group cooperation (1)
		Positive social experience (1)
	Relatedness with the student teacher	Positive perception of the student teacher (4)
		Positive student teacher - pupil relationship (1)
Competence	Appropriate task difficulty	Choosing easier tasks to support competence (2)
	Increased learning effectiveness	Learning progress (2)
		Improved performance during the concert (1)
		Sense of mastery (2)
	Overcoming obstacles	Reducing blocks in creativity (1)
	Improved quality of performance	Development of skills (1)
Amotivation	Decline after the end of the project	Return to old patterns (1)
	Withdrawal due to negative conditions	External discomfort (1)
Attitude change	Positive change in motivation	Increased interest in music (1)
		Reflection and decision to continue (1)
	Development of self-initiative	Increased self-initiative (1)
		Learning through collaboration (1)
Enthusiasm	Increased energy for learning	General increase in enthusiasm (1)
	Active engagement	Self-initiated use of learning materials (1)
Appealing repertoire	Positive reception of the repertoire	Enjoying the repertoire (1)

**Table 4:** Observed changes in pupils’ motivation in music theory lessons by their music theory teachers.

Theme	Category	Code
Autonomy	Independence in written assessment	Lack of independence in written assessment (1)
Competence	Improved academic performance	Better assessment results (2)
		Increases in understanding (1)
	Mastery of the subject	Positive progress (3)
		Faster conceptual connections (1)
	Perceived efficacy	Increased self-confidence (1)
Attitude change	Increased classroom participation	More participation (3)
		Increased activation (1)
	Change in approach	Change in working habits (2)
	Interest in the subject	Increased interest in the subject (2)
	Attitude toward the class	Improved attitude (1)
	Change in motivation	More motivation (1)
		More initiative (1)
	Responsibility	Class readiness (1)
	Response to encouragement	Positive reactions to the encouragement (1)

The most prominent theme observed by the instrument teachers was increased *relatedness among peers* and *with the student teachers.* We report the emerging themes in order of appearance as in our research question:

Increased *autonomy* was observed, as a display of *intrinsic motivation*, as some pupils started to practice without external prompting, as noted by a teacher: “Increased interest in practicing at home, that he starts on his own without me having to push him.”

The instrumental teachers observed the greatest increase in *relatedness*. The theme relatedness appears in two categories: *relatedness among peers* and *relatedness with the student-teacher*.

The code *positive social experience* reflects the statement: *“This social aspect. I think it brightened up their school day.”* The increased connection among pupils depicts the codes: *physical closeness and shared activity, peer bonding, and new friendships.* The pupils began to enjoy the musical activities, which encouraged them to connect with their peers. They enjoyed the experiences of *peer support* as described by the teacher: *“He liked that he was not accompanied by a teacher, but by his peer.”* Music-making became a democratically shared activity by all pupils. The code *group cooperation* further enhances the effect of increased relatedness that one of the teachers described: *“They became even more motivated for making music together and for the big performance.”*

The code *relatedness with the student teacher* was very prominent, as pupils frequently described their student teachers to their instrumental teachers, as depicted in the quote: “*All three were very enthusiastic about the teacher.”* The pupils had a very positive perception of their student teacher and had positive relationships: *“He got along very well with the student.”*

Instrumental teachers reported increased *competence*, and their observations fell into four categories: *appropriate task difficulty, increased learning effectiveness, overcoming obstacles,* and *improved performance quality.*

The code *choosing easier tasks to support competence* was used to depict instrumental teachers’ observations that the repertoire at the workshops was easier compared to their usual music school curricula: *“I have to say that because the piece was easier, it was also more manageable for him.”* Throughout the intervention, teachers noticed *learning progress*, *improved performance during a concert,* and a greater *sense of mastery* in their pupils: *“After these practice sessions (Great practitioner), she did everything really well.”* Under the guidance of student teachers, pupils felt more competent even at tackling more complex tasks, i. e. improvisation, and *developed their skills*: *“And it is very noticeable in the playing (the learned patience and precision).”*

Some pupils exhibited *amotivation*; although they were motivated during the project, their motivation *declined after its end,* and they *returned to their old patterns,* as noted by a teacher: *“Ever since this project ended again, it’s the same old story.” One* student *withdrew due to negative conditions,* as they felt *external discomfort: “It was too loud, and she decided she would not continue.”*

Attitude change fell into two categories: *positive change in motivation* and *development of self-initiative.* Teachers observed their pupils’ *increased interest in music: “Just a lot of interest in this area. She already had a big desire before, and now it has become even bigger.”* Some pupils were enc*ouraged to reflect and change their decision to drop out*: *“She had already thought about quitting, but she changed her mind and wants to continue.”*

Pupils’ *self-initiative developed* and increased, especially as they started to *learn through collaboration,* as noted by one of the teachers: *“It’s great that she had someone to work with, because she then learned patience.”*

Enthusiasm was exhibited in *increased energy for learning* and *active engagement.* The *general increase in enthusiasm* is best depicted in a teacher’s statement: *“There was more drive in general.”* Some pupils started to use learning materials at home: *“She herself puts the sheet music on the stand—what they worked on in the workshop—and then we continue working on it a bit during the lesson.”* Teachers observed that appealing repertoire at workshops contributed to greater motivation. Pupils *enjoyed the repertoire,* as noted by a teacher: *“They really liked the repertoire.”*

The most prominent theme observed by the music theory teachers (Table 4) was increased attitude change. We report the emerging themes in order of appearance in our research question.

Music theory teachers rarely observed a lack of autonomy in some pupils, especially in their independence in written assessments, while they did not observe any changes in relatedness.

A much more prominent theme was increased competence, where music theory teachers’ observations fell into three categories: *improved academic performance, mastery of the subject,* and *perceived efficacy.* The category *improved academic performance* consists of *better assessment results* and *increases in understanding* codes, which one of the teachers described: *“Compared to the first semester, she has really been diligent in the second. She also wrote the test and got a 4.”* The pupils were observed to have increased their listening comprehension, as described by the teacher: *“Also regarding listening comprehension and practical as well as theoretical knowledge, she did really well.”* The instructional support by the student teachers helped pupils toward *mastery of the subject, as evidenced by code-positive progress* and *faster conceptual connections, as* described by a teacher: *“You can also feel progress in the sense that during explanations they connect concepts a bit faster.”* The last category is *perceived efficacy, in which* music theory teachers observed increased pupils’ self-confidence, as one teacher described: *“She is more confident when solving tasks, but she still struggles a lot, mixing things up.”*

The most prominent theme, increased attitude change, fell into five categories: *increased classroom participation, change in approach, increased interest in the subject, changed attitude toward class,* and changes *in motivation, responsibility,* and *response to encouragement.*

**Figure 1:**
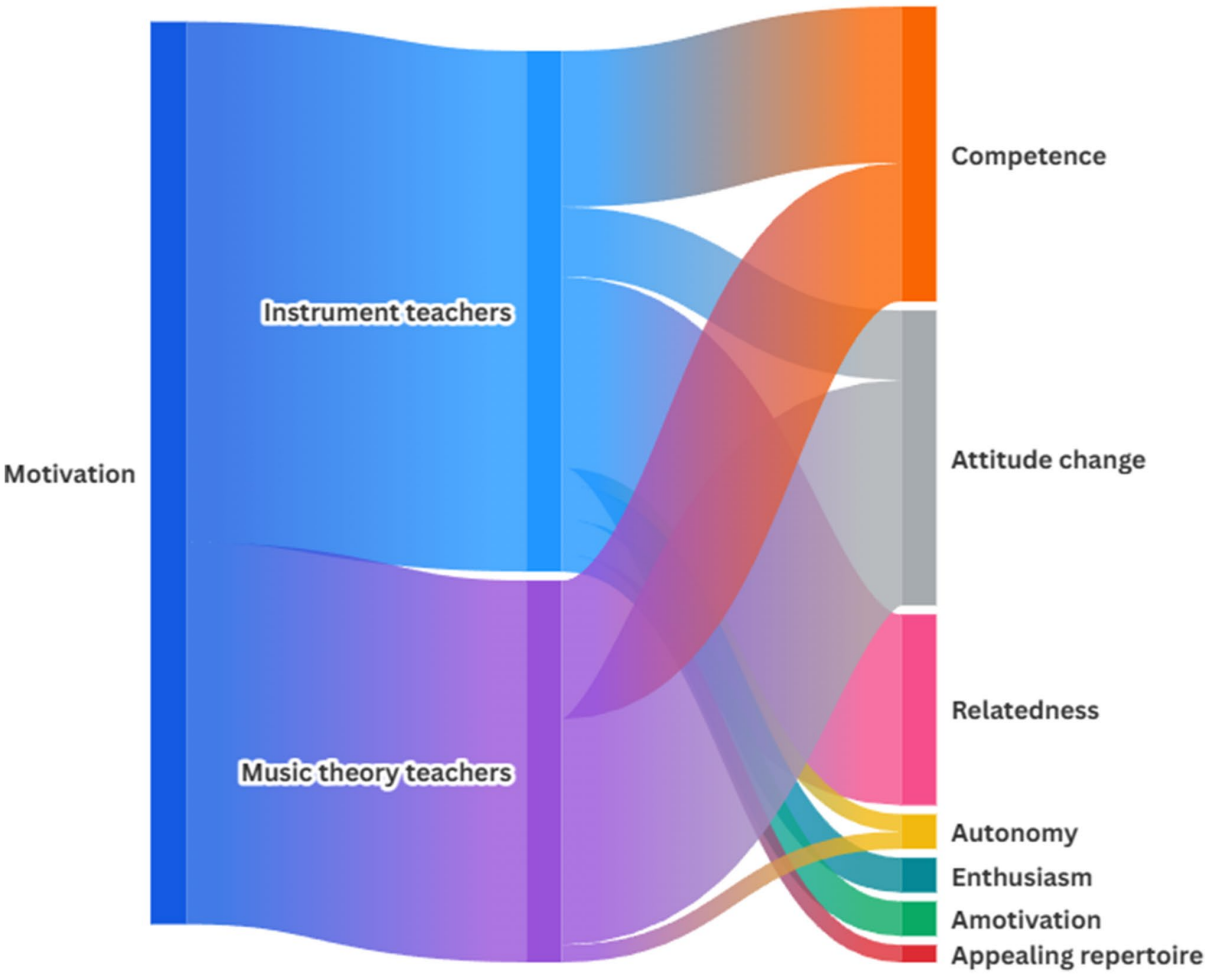
The Sankey diagram 1: comparison of observed changes in pupils’ motivation between music instrument and music theory teachers.

The code reflects the statement: *“With some of them I noticed more participation… all three showed more participation.” Pupils* were observed to have *increased activation,* as one teacher described: *“P1 has actually become a bit more activated, I have to say.”* Pupils started to show more *interest in the subject,* a *changed attitude toward the class,* and also positive *changes in motivation,* where they exhibited *more motivation and more initiative,* as one of the music theory teachers noted: *“Maybe the* [Super Music Theory] *was an encouragement that made her start working a bit more on her own.”* Pupils were observed to show greater *responsibility,* as evidenced by the code class readiness. One of the teachers noted*: “They are prepared for lessons, they come with their homework done.”* Music theory teachers observed that pupils responded more positively to encouragement, as one teacher noted: *“The boys, who usually hide their insecurity behind inappropriate behavior, respond noticeably faster and more positively to my encouragement (Figure 1).”*

The Sankey diagram compares observed changes in pupils’ motivation reported by two groups: music instrument teachers and music theory teachers, following the intervention. Instrument teachers identified motivational changes across seven categories, suggesting a broader, more varied perception of change. According to their observations, most substantial observed shifts were in relatedness, with significant increases also in competence and attitude change, alongside smaller effects in autonomy, enthusiasm, amotivation, and appealing repertoire. In contrast, music theory teachers noted changes in far fewer categories, reflecting a narrower scope of their observations in pupils. Within these, attitude change toward music theory was most prominent, with more modest increases in competence, and the least in autonomy.

In the subsequent school year 2025/2026, of the 55 pupils who participated in the intervention, 3 did not enroll; the others continued their musical education at the local public music school.

## Discussion

In the quantitative part of our study, we observed statistically significant changes in two of the five measured constructs. Perceived autonomy in theory lessons increased after the intervention, and perceived competence in instrument playing decreased. Statistically significant findings were supported by effect sizes ranging from moderate to strong, suggesting that the applied intervention had a measurable effect on pupils. However, due to the limitations of this study, these results should nonetheless be interpreted with caution, and no definitive conclusions about the precise magnitude of the intervention effects can be drawn.

Since music theory pupils’ perceived *autonomy* increased after the *I Can Master Music!, Super Music Theory!,* and *Connected* interventions, the data suggest that these interventions were partially successful. However, as music theory lessons are among the significant factors in dropout (Kavčič Pucihar et al., 2023; Kavčič Pucihar et al., 2024; Sagrillo, 2016), this may be an essential finding. The music theory workshops in the intervention used learner-centered teaching approaches, moving away from the traditional format in music theory lessons. We intertwined the subject’s fundamental musical activities with playful approaches and activities that moved away from traditional, teacher-centered teaching methods. The activities included musical games, body movement, and creativity, with improvisation on Orff musical instruments and pupils’ chosen instruments. As observed by the students leading the workshops, pupils’ motivation to participate in the activities diminished as soon as the content approached specific music theory, such as listening to and defining musical intervals. The pupils’ feedback led the students to choose the music activities as their incentive, moving their teaching approach toward a more learner-centered approach, confirming that teachers’ instructional strategies are critical for enhancing students’ motivation (Kiss et al., 2025). Our findings align with a Slovenian study by Smolej Fritz and Peklaj (2011), which found that students who perceive music theory as useful demonstrate greater competence and motivation, whereas those who fear the subject feel less competent and less motivated. As Cogdill (2015) identifies SDT as a useful framework for explaining how learner-centered music programs that are responsive to students’ psychological needs can promote both persistence and transfer of motivation across contexts, our findings also align with Fasano et al. (2025), where learner-centered orchestral training fostered more internalized forms of motivation in children, also in other areas of their academic performance.

Our preliminary exploratory study results showed no changes in *relatedness* across the groups, suggesting that this basic psychological need is more stable and would require a different approach to make possible changes measurable.

The pupils’ decreased perceived *competence* in instrument playing can be explained by the Dunning-Kruger (Kruger and Dunning, 1999) effect. This effect states that people tend to hold overly favorable views of their abilities in many social and intellectual domains, including education. The authors suggest that this overestimation occurs, in part, because people who are unskilled in these domains suffer a dual burden: not only do they reach erroneous conclusions and make unfortunate choices, but their incompetence robs them of the metacognitive ability to recognize it. Yang Hansen et al. (2024) confirmed the Dunning-Kruger effect in mathematics among pupils in 3rd and 4th grade across all six European countries involved in the research. Their findings indicate a general susceptibility to this phenomenon.

The workshops for the instrumentalists in the intervention were aimed at improving pupils’ competence also through guided practice and metacognitive strategies, which, as Benton (2013) suggests, all contribute to improved metacognitive skills that in turn may have contributed to pupils’ greater awareness of their relatively low competency level in playing their instrument. With increased awareness of their competence levels, pupils may have discovered that their competence in playing a musical instrument is not as high as they thought pre-intervention, thereby confirming the Dunning-Kruger effect. The observations of their musical instrument teachers - which we present in detail in the qualitative part of the discussion - confirm that their actual competence level in instrumental performance improved after the intervention. Since Hewitt (2010) discovered that instruction in self-evaluation had little impact on students’ self-evaluation accuracy or music performance, we decided to include pupils’ instrument and music theory teachers in the qualitative part of our study to obtain a more in-depth estimation of their overall musical development, with special emphasis on observed possible changes in motivation.

Our preliminary exploratory study results showed no changes *amotivation* across the groups.

Since the literature review revealed no quantitative studies investigating interventions to reduce music-school dropout, we cannot compare our preliminary quantitative findings with similar studies.

To gain a broader and deeper understanding of the quantitative findings, qualitative data from music instrument and theory teachers were obtained and analyzed. In the qualitative part of the study, in focus group interviews, we asked music instrument and music theory teachers about their observations of changes in their pupils’ motivation resulting from participation in the intervention.

### Music instrument teachers

Music instrument teachers identified motivational changes across seven themes, as depicted in Table 3. The most prominent changes occurred in relatedness, with significant increases also in competence and attitude change, alongside smaller effects in autonomy, enthusiasm, amotivation, and increased motivation due to appealing repertoire. In contrast, music theory teachers noted changes in fewer themes, as depicted in Table 4, reflecting a narrower scope. Among these, attitude change toward music theory was most prominent, with more modest increases in competence and autonomy.

The discussion is structured around seven themes that emerged from the content analysis, first in the context of music instrument teachers and, second, in the context of music theory teachers.

Musical instrument teachers identified four themes that align with the SDT theory as proposed by Ryan and Deci (2002): autonomy, relatedness, competence, and amotivation. These core constructs provide a foundation for understanding motivation also in the context of instrumental music education. There are numerous studies that study motivation in instrumental music education (Comeau et al., 2015; Evans et al., 2013; Evans and Liu, 2019; Földi and Jozsa, 2022; Földi et al., 2024; Gerelus et al., 2020; Kavčič Pucihar et al., 2024; Schatt, 2018; Schatt, 2022; Utomo, 2025; Wieser et al., 2024; Wieser and Müller, 2025) within this theoretical framework. Psychological need satisfaction is strongly associated with intentions to continue playing a musical instrument (Evans and Liu, 2019). Some pupils in our study were observed to exhibit amotivation, according to Deci and Ryan (2002), one of the three major forms of motivational regulation. This result was consistent with Kavčič Pucihar et al. (2024) study, in which, among the reasons for dropout, they identified loss of interest, demotivation, and boredom. Gerelus et al. (2020) also found that dropout students exhibited less autonomous motivation and stronger amotivation.

Kavčič Pucihar et al. (2024) reported that deficiencies in relatedness, competence, and autonomy contribute significantly to student dropout. Consequently, the outcomes of the present intervention—namely teachers’ perceptions of enhanced relatedness among pupils and with the student teacher, alongside observed gains in competence and autonomy—suggest a positive effect. The theme attitude change aligns with one of the central tenets in the motivational theory of planned behavior (Ajzen, 1991; Ajzen, 2011). Observations of the instrumental teachers in our study align with Yoo’s (2020) study, which emphasized that students with a positive attitude toward and a value for music are more likely to continue participating in musical activities even when faced with obstacles. As over-emphasis on music theory and solfège, unappealing repertoire, and little room for creativity and performance dampen enthusiasm (Kavčič Pucihar et al., 2024), our study revealed increased enthusiasm in pupils as observed by the music instrument teachers. The support from the student teachers, especially through positive encouragement, yielded results similar to those of Qi et al. (2021), which aimed to heighten enthusiasm, thus fostering a better attitude and music performance. Music instrument teachers observed that appealing repertoire contributed to pupils’ motivation. Student teachers invited pupils to participate in the repertoire selection. From Weiner’s Attribution Theory (1972) standpoint, giving pupils a role in selecting their repertoire increases their perceived control over the learning process. By attributing their engagement and progress to choices they can influence, pupils view the cause as internal and controllable rather than externally imposed, which enhances their motivation. Our findings align with Renwick and McPherson (2002) and Šulentić Begić et al. (2024), who found that when pupils have choice over what they play, they practice more, persist longer and engage more deeply.

### Music theory teachers

The music theory teachers reported a remaining lack of *autonomy*, no changes in *relatedness,* and improved *competence*, compared to pre-intervention. Both observed changes are covered in SDT (Deci and Ryan, 2002). Kavčič Pucihar et al. (2024) identify the absence of competence and autonomy as factors contributing to dropout. Even though a lack of autonomy remained evident among some pupils, it is important to acknowledge that the intervention increased pupils’ observed competence in music theory classes.

The most prominent theme observed by the music theory teachers (Table 4) was increased attitude change. This finding aligns with the theory of planned behavior (Ajzen, 1991, 2011). That theory posits that attitude change is a major contributor to motivated behavior. In the workshops *I Can Master Music!, Super Music Theory,* and *Connected,* student teachers encouraged pupils. They used learner-centered strategies that engaged pupils. As a result, the pupils were much more active and involved than in their usual music school theory classes. Our findings align with Ruybalid (2016), who found that students’ attitudes toward general music classes were a significant predictor of persistence in music activities. The observed change in attitude toward music theory classes, as described by the music theory teachers, is promising.

### Integration of qualitative and quantitative findings

We used a mixed-methods approach to provide a more comprehensive picture of the potential effects of the intervention on pupils’ motivation, drawing on pupils’ self-reports and instrumental and theory teachers’ observations. Overall, the two strands of data showed several divergences across the SDT constructs of relatedness, autonomy, competence, and amotivation. For relatedness, quantitative analyses showed no statistically significant changes, whereas musical instrument teachers described increased relatedness among pupils. For autonomy, quantitative data indicated a statistically significant increase in music theory pupils, yet one music theory teacher reported persistently low autonomy during written examinations. Conversely, although no statistically significant changes in autonomy were observed among music instrument pupils, one instrument teacher reported greater autonomy in independent practice without external prompting. Regarding competence, teachers of music theory pupils reported increased competence after the intervention, despite no corresponding quantitative change. In contrast, pupils’ self-reported competence in instrumental learning decreased, while instrumental teachers reported marked improvements in pupils’ instrumental performance competence. Finally, although some instrument teachers reported amotivation in specific pupils, this was not reflected in pupils’ self-reports. These divergences may reflect measurement limitations (see Methodological limitations), differences in sampling (quantitative analyses included only pupils who completed both questionnaires), and differences in perspective and context (pupil self-reports vs. teacher observations across lessons). Importantly, qualitative findings supplemented the quantitative results by highlighting attitude change across both teacher groups. Instrument teachers also described increased enthusiasm and motivation, which they partly attributed to the use of appealing repertoire.

Our intervention supplemented the usual music school curriculum. The activities in the workshops were new and interesting to the music school pupils, especially since they had University students as their teachers.

This was the first intervention in the Slovenian music school system, aimed at reducing dropout by enhancing pupils’ motivation through support for their basic psychological needs for competence, autonomy, and relatedness, as defined in Self-determination theory (Deci and Ryan, 2002), within the ongoing music-teaching process. Hence, there is no recorded data to directly compare the results of our intervention. Our exploratory study applied mixed methods. The use of both qualitative and quantitative data contributed to a broader understanding of the possible underlying mechanisms of the applied intervention. While qualitative data provide additional context to quantitative results, no definitive conclusions can be drawn due to the study’s limitations.

## Limitations

Our intervention aimed to reduce dropout in music at a local music school and lasted 8–10 45-min sessions over 4 months, which is a relatively short period for an intervention. The sample of pupils was relatively small. At the beginning, there were 55 pupils; at the end, 43 pupils also completed the 2nd questionnaire after the intervention. The results showed changes in pupils’ motivation following the support of their basic psychological needs, which may have influenced their intention to remain in music school, however, we did not have a control group to compare the effect of our intervention on the drop-out itself. One needs to bear in mind that there were student teachers working with pupils, not professional teachers, which might have altered the results.

## Methodological limitations

As we conducted multiple statistical tests, the risk of false-positive findings increased. No formal adjustment for multiple comparisons was applied, and the reported *p*-values should therefore be interpreted with caution. This limitation may affect the robustness of individual statistically significant results. This is why it is important to note that no concrete conclusions can be drawn. Another important limitation is that the 2nd questionnaires were administered immediately after their final concert, and pupils’ emotional arousal might have been altered from usual levels. Pupils were relatively young and therefore had trouble with self-evaluation and understanding the questionnaires, although they received help from the questionnaire administrators. Given the substantial number of variables in our study, the absence of additional statistically significant results provides an incentive for further research to achieve a more comprehensive and nuanced understanding of the underlying mechanisms of our interventions. The absence of statistically significant results can be attributed to a relatively small sample of 43 pupils. The sample size can greatly affect the outcomes of hypotheses and the conclusions drawn from the interpretation of results (Serdar et al., 2021). Like many educational research studies, our study also recruited a small number of participants, which, in turn, reduced the statistical power of our results, even though the possibility of large and important differences remains (Cook and Hatala, 2015). Another limitation of our study is that we did not validate the adapted questionnaires, which may have influenced our results. We were unable to find an original questionnaire that would measure all the researched concepts within the music schools, which is why we have decided to adapt the scales and items.

## Conclusion

The findings of our exploratory study, rooted in SDT (Deci and Ryan, 2002), show increased perceived autonomy in music theory pupils and decreased perceived competence in music instrument pupils after the intervention (quantitative results). The qualitative data diverge from the quantitative data, presenting music teachers’ observations of pupils, and situating these results within the music education process. Observations by musical instrument teachers revealed increased competence, relatedness, and autonomy, as well as amotivation in pupils. They also noticed a positive change in attitude, more enthusiasm, and greater motivation, specifically attributed to the appealing repertoire. Music theory teachers observed increased competence among pupils, while one case showed no change in autonomy. The most prominent observed change was a positive attitude toward music theory lessons. Our exploratory study is the first of its kind in the Slovenian context. It offers initial insights into potential changes in motivation when pupils’ needs for autonomy, relatedness, and competence are supported in their music education, before dropout decisions are made.

Due to the limitations described, the study’s findings cannot be generalized to all public music schools in Slovenia or internationally. However, this study is an exploratory step toward proactively reducing dropout in Slovenian music schools.

### Future implications

The themes of competence, attitude change, and relatedness bring an important message to professional music educators. These findings highlight the importance of supporting pupils’ perceived competence in the learning process, as this is reflected in changes in students’ attitudes. Furthermore, these findings show that music educators must be masters at creating meaningful interpersonal relationships with their pupils, thus bringing valuable learning material for present and future teachers’ education. Slovenian music school teachers should receive adequate instructional support to develop effective teaching approaches for the youngest musicians - music school pupils. A follow-up study is needed to properly evaluate the intervention’s long-term outcomes. With lessons learned, a similar intervention could be implemented at another Slovenian or other music school. Given that attitude change, enthusiasm, and appealing repertoire were categories highlighted by the music school teachers, it would seem sensible to explore the findings within other psychological theories. Comparing the obtained findings would provide even more valuable insights into ways to successfully address, and ideally reduce, dropout in Slovenian music schools and internationally.

## Data Availability

The original contributions presented in the study are included in the article/supplementary material, further inquiries can be directed to the corresponding author.

## Appendix 1

### Descriptive statistics of included variables

**Table:**

Variable	M	SD	Min	Max	A	K	Shapiro–Wilk	
							Statistic	*p*
Age	10.37	1.83	7	15	0.01	−0.50	0.94	0.02
Intrinsic motivation (INS) pre-intervention	31.21	4.06	21	35	−0.88	−0.36	0.85	< 0.001
Intrinsic motivation (INS) post-intervention	31.18	4.57	19	35	−1.01	0.01	0.83	< 0.001
Amotivation (INS) pre-intervention	7.23	3.80	5	23	2.24	6.15	0.62	< 0.001
Amotivation (INS) post-intervention	8.40	5.99	5	29	2.32	5.13	0.61	< 0.001
Intrinsic motivation (MTL) pre-intervention	21.10	10.22	18	42	−0.09	0.70	0.91	0.008
Intrinsic motivation (MTL) post-intervention	22.27	10.10	5	35	−0.32	−1.16	0.65	< 0.001
Amotivation (MTL) pre-intervention	14.65	9.42	5	35	0.63	−0.83	0.87	0.001
Amotivation (MTL) post-intervention	17.25	10.70	5	35	0.28	−2.32	0.88	0.002
Autonomy (INS) pre-intervention	30.07	6.92	18	42	−0.09	0.72	0.95	0.15
Autonomy (INS) post-intervention	28.95	6.71	15	42	−0.13	−0.61	0.97	0.44
Competency (INS) pre-intervention	29.76	5.26	10	35	−1.94	4.85	0.77	< 0.001
Competency (INS) post-intervention	26.36	4.09	15	35	−0.46	0.63	0.91	0.01
Relatedness (INS) pre-intervention	28.86	6.14	12	35	−0.96	0.23	0.89	0.003
Relatedness (INS) post-intervention	29.50	4.09	10	35	−1.29	1.19	0.83	< 0.001
Autonomy (MTL) pre-intervention	17.61	6.19	6	36	0.86	0.72	0.89	0.002
Autonomy (MTL) post-intervention	21.11	7.78	9	42	0.39	2.70	0.93	0.04
Competency (MTL) pre-intervention	23.02	7.12	7	35	−0.46	−0.56	0.95	0.25
Competency (MTL) post-intervention	23.24	6.67	9	35	−2.24	0.70	0.95	0.11
Relatedness (MTL) pre-intervention	27.07	7.74	6	35	−0.87	1.04	0.90	0.004
Relatedness (MTL) post-intervention	28.40	6.73	9	35	−1.01	2.70	0.92	0.02
